# The Roles of Noncardiomyocytes in Cardiac Remodeling

**DOI:** 10.7150/ijbs.47180

**Published:** 2020-07-02

**Authors:** Dan Yang, Han-Qing Liu, Fang-Yuan Liu, Nan Tang, Zhen Guo, Shu-Qing Ma, Peng An, Ming-Yu Wang, Hai-Ming Wu, Zheng Yang, Di Fan, Qi-Zhu Tang

**Affiliations:** 1 Department of Cardiology, Renmin Hospital of Wuhan University, Wuhan 430060, RP China.; 2 Cardiovascular Research Institute of Wuhan University, Wuhan 430060, RP China.; 3 Hubei Key Laboratory of Metabolic and Chronic Diseases, Wuhan 430060, RP China.; 4 Department of Thyroid and Breast, Renmin Hospital of Wuhan University, Wuhan 430060, RP China.

**Keywords:** cardiac remodeling, noncardiomyocytes, cardiac fibroblast, immune cells

## Abstract

Cardiac remodeling is a common characteristic of almost all forms of heart disease, including cardiac infarction, valvular diseases, hypertension, arrhythmia, dilated cardiomyopathy and other conditions. It is not merely a simple outcome induced by an increase in the workload of cardiomyocytes (CMs). The remodeling process is accompanied by abnormalities of cardiac structure as well as disturbance of cardiac function, and emerging evidence suggests that a wide range of cells in the heart participate in the initiation and development of cardiac remodeling. Other than CMs, there are numerous noncardiomyocytes (non-CMs) that regulate the process of cardiac remodeling, such as cardiac fibroblasts and immune cells (including macrophages, lymphocytes, neutrophils, and mast cells). In this review, we summarize recent knowledge regarding the definition and significant effects of various non-CMs in the pathogenesis of cardiac remodeling, with a particular emphasis on the involved signaling mechanisms. In addition, we discuss the properties of non-CMs, which serve as targets of many cardiovascular drugs that reduce adverse cardiac remodeling.

## Introduction

Cardiac remodeling refers to structural and functional abnormalities of the heart (mainly left ventricular, LV) that develop in response to various internal or external pathological stimuli. Cardiac remodeling often causes changes in the wall thickness, ventricular volumes and cardiac mass, which further leads to reduced ventricular ejection and imbalances in the neurohumoral system, this, in turn, aggravates ventricular remodeling and ultimately causes noncompensatory heart failure (HF). Cardiac remodeling is a remarkable pathogenic manifestation of many serious cardiovascular diseases that ultimately progress to HF 1-5. Studies have shown that structural, electrical and energetic remodeling are involved in this progression, among which the former two play a leading role 6. Cardiac remodeling is closely related to the prognosis of clinical HF and has become an important therapeutic target for HF 7.

The heart is a complex multicellular tissue consisting of a heterogeneous population of CMs and morphologically and functionally distinct non-CMs (including fibroblasts, immune cells, pericytes and endothelial cells), with CMs accounting for approximately 22.8% of the total population and non-CMs accounting for the rest 8. Cardiac remodeling is well regulated by cell apoptosis, proliferation, migration and differentiation in cardiac or extracardiac tissues. A recently published transcriptome profiling study of 21422 single cells collected from normal and failing heart samples revealed the important roles of non-CMs in regulating the behavior of CMs 9. Numerous studies have reported that non-CMs contribute to various heart diseases, cardiac regeneration and the initiation and development of cardiac remodeling 10, 11.

This review summarizes the current understanding of the involvement of non-CMs in the pathogenesis of cardiac remodeling, with a particular emphasis on the potentially involved regulatory factors and signaling mechanisms. In addition, the properties of non-CMs are discussed, since they are important targets of many cardiovascular drugs that alleviate adverse cardiac remodeling.

## Cardiac fibroblasts

The adult mammalian heart is enriched in fibroblasts. Cardiac fibroblasts (CFs) account for approximately 27%, 64%, and 72% of the heart mass in mice, rats and humans, respectively 12. Previous studies have shown that CFs are a significant cellular effector involved in cardiac remodeling, especially in the regulation of cardiac fibrosis. When exposed to pressure/volume overload or other pathological stimuli, CFs will differentiate into myofibroblasts, which can produce large amounts of extracellular matrix (ECM) proteins. However, the significance of CF extends far beyond regulating the production of ECM. Instead, CFs play a critical role not only in maintaining structural integrity and systolic function of the infarcted zones via the formation of matrix components within a relatively short time, but also in causing abundant collagen deposition and cardiac fibrosis due to their constant response to pathophysiological stimuli. In the past, due to the lack of CF-specific markers, many facts about CF biology remained controversial. With the generation of genetic mouse models that express the CF-specific Cre recombinase 13-15, it is possible to know more about this cell type.

### Origins of cardiac fibroblasts

There are several major sources of CFs, among which resident CFs remain the predominant source. Experiments using genetic lineage tracing approaches have shown that the resident fibroblast lineage was derived from epicardium-derived progenitor cells (EPDCs) undergoing epithelial-to-mesenchymal transition (EMT) during embryonic development 16. During the development of the heart, EPDCs migrate to the myocardium and are trapped in the interstitium of the myocardium and finally become resident CFs. During EPDC mobilization, the cooperation of myocardin-related transcription factor (MRTF) and serum response factor (SRF) via the MRTF-SRF axis is required 17. Another study performed with fluorescence-based genetic lineage tracing technology revealed that the post-MI scar principally collected epicardium-derived myofibroblasts, which could be specifically ameliorated by deletion of the Mrtf or Srf genes, indicating that resident CFs mainly originate from the epicardium under pathological conditions 18. Additionally, it has been reported that a small fraction of CFs residing in the heart have an endothelial origin 19, 20. Epithelial- and endothelial-derived cells altogether contribute to the formation of resident CFs. Interestingly, after undergoing pressure overload injury, CFs from different embryonic sources exhibited almost the same phenotypes and gene expression modes, suggesting the possibility that CF proliferation is affected by the cellular microenvironment instead of the developmental origins 19.

**Figure 1:**
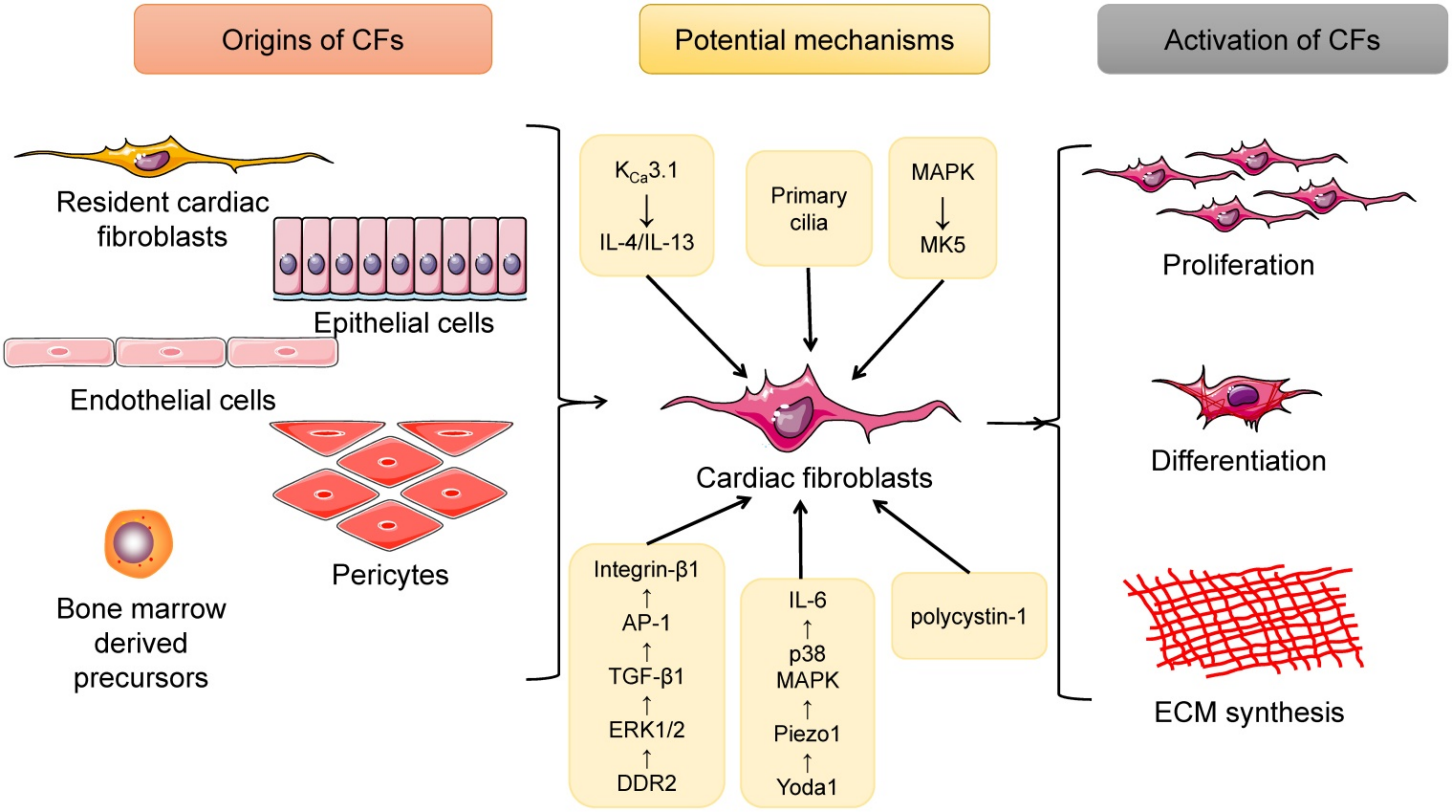
Summary of the origins as well as the activation of cardiac fibroblasts (CFs). CFs have several sources, including resident cardiac fibroblasts, epithelial cells, endothelial cells, pericytes, and bone marrow-derived cells. When exposed to pressure/volume-overload or other pathological stimuli, CFs will undergo proliferation as well as differentiation into myofibroblasts, cells that can produce large amounts of extracellular matrix (ECM) proteins and directly contribute to cardiac fibrosis. In addition, a lot of potential mechanisms are involved in this process.

In addition to resident CFs, some other extracardiac cell sources are contributors to the expanding myofibroblast population in the remodeled heart. These include endothelial cells, pericytes and bone marrow-derived cells (BMCs). Endothelial cells undergoing endothelial-mesenchymal transition (EndMT) eventually transform into CFs, the process of which can be induced by transforming growth factor-beta 1 (TGF-β1) and blocked by bone morphogenic protein 7 (BMP-7) 21. Moreover, Gli1-positive perivascular MSC-like cells proliferated after heart injury, and depletion of these cells protected against postinfarct remodeling 22. Investigators also found CD45^+^/eGFP^+^ inflammatory cells 23 and monocyte-derived myofibroblasts 24 in the infarcted heart. Furthermore, in a cardiac fibrosis rat model induced by angiotensin II (Ang II) infusion, bone marrow-derived monocytes were found to differentiate into myofibroblasts, which involved the interaction of the K_Ca_3.1 and TRPV4/TRPC6 channels 25. Additionally, cardiac macrophages were tracked in LysM(Cre^/+^);ROSA26(EYFP^/+^) transgenic mice undergoing MI surgery, and both *in vivo* and *in vitro* experiments indicated that macrophages were capable of differentiating into a fibroblast-like phenotype during myocardial healing after MI 26. Therefore, CFs are a cell type with multiple sources, among which resident CFs account for the majority of CFs, and BMC-, epithelial-, endothelial- and pericyte-derived CFs are also valuable (Figure 1).

### Mechanisms underlying CF activation in cardiac remodeling

It is widely acknowledged that CF is a key mediator that regulates the integrity and function of infarcted heart tissues by secreting ECM and some regulatory factors, and a variety of signaling pathways are involved. Many of the latest studies have revealed new insights into the regulatory roles of CFs.

CF activation is an extremely complex biological process. The data obtained from CF stage-specific lineage tracing provided an in-depth understanding of the differentiation states and dynamics of CFs in the process of scar formation following MI. Resident CFs in infarcted regions usually progressed through the following three stages: in the early stage (within 2-4 days), CFs were maximally activated and expanded in number by 3.5-fold; in the middle stage (days 4-7), CFs differentiated into myofibroblasts that secreted large amounts of ECM to maintain the integrity of the heart tissue; and finally (by day 10), these cells gradually lost the ability to proliferate and produce α-SMA and ultimately turned into matrifibrocytes during scar maturing 27.

Additional evidence from current studies has shown that a range of genes, molecules, and even cellular structures are involved in CF proliferation and activation in response to physiological and pathological factors 28-32 (Figure 1).

Recent research utilized two- and three-dimensional (2D vs 3D) culture conditions to study the topological arrangement of CF after heart injury, and the authors found that the recruitment, proliferation, and aggravation of CFs to the injured area induced gene expression changes, chromatin remodeling, and altered phenotypic features during the healing of injury 31.

It has been well established that many ion channels are important mediators of CF activation, among which Ca^2+^ channels are of great importance 33-35. K_Ca_3.1 channels facilitated cardiac remodeling mainly by exacerbating cardiac fibrosis as well as inflammation; K_Ca_3.1 channels promoted the secretion of interleukin-4 (IL-4) and IL-13, both of which upregulated the differentiation of bone marrow-derived monocytes into fibrocytes, which are cells that mature into fibroblasts and eventually myofibroblasts; K_Ca_3.1 channels accelerated the infiltration and differentiation into macrophages of monocytes, which further induced inflammation in the heart 25. Another very recent study provided evidence that Piezo 1, a Ca^2+^-permeable ion channel, was highly expressed and played key roles in murine and human CFs. The mechanical activation of Piezo1 channels was specifically induced by Yoda 1, and then IL-6 expression was enhanced via the p38 mitogen-activated protein kinase (MAPK) pathway 30. The MAPK signaling pathway is another effector, of great pathophysiological importance, for the migration, proliferation and differentiation of CFs. MAP kinase-activated protein kinase 5 (MK5) is a protein serine/threonine kinase that is activated by both the p38α/β MAPKs and the atypical MAPKs ERK3 and ERK4. CFs isolated from MK5^-/-^ and MK5^+/-^ mice exhibited decreased secretion of type 1 collagen and fibronectin compared with those isolated from MK5^+/+^ mice, while MK5-deficient fibroblasts were showed reduced contraction 32. Recently, studies have also indicated the role of collagen receptor cross-talk in cardiac remodeling: cross-talk between DDR2 and Integrin-β1 influences collagen type I and α-SMA expression in Ang II-stimulated CFs via ERK1/2 MAPK-dependent TGF-β1 signaling 28.

Cellular structures can also modulate the cardiac remodeling process. In injured myocardium stimulated by disease-related stress, a cellular microstructure named the primary cilium, which is harbored in CFs from both neonatal and adult hearts, was first discovered. Primary cilia along with polycystin-1 (PC1) were first recognized as crucial regulators of TGF-β1-induced cardiac fibrogenesis. PC1 knockdown in myofibroblasts significantly increased adverse cardiac remodeling following MI 29. Taken together, the results of these studies suggest that CFs modulate cardiac remodeling at various levels via cells, proteins and genes. Importantly, an in-depth investigation is required to systematically reveal the effects of CFs.

### Therapies targeting CFs

Since the mechanisms involved in CF activation have already been demonstrated, therapies targeting CFs seem to be promising, and their implementation has progressed from the bench to the bedside. A very recent study demonstrated PET imaging of activated CFs by utilizing a ^68^Ga-labeled fibroblast activation protein (FAP) inhibitor (^68^Ga-FAPI-04), which was a noninvasive and repeatable technique that provided new insights into the diagnosis and prognosis of heart diseases (such as post-MI syndrome, cardiac fibrosis, hypertension, HF, and other conditions) related to CF activation 36. Revascularization of the affected cardiac tissue appears to be a promising strategy to attenuate the effects of adverse cardiac remodeling. Delivery of growth factors (VEGF, PDGF, and bFGF) into injured heart tissue has increasingly been used 37, 38. However, challenges related to low efficacy, a high loss rate and low retention remain to be solved. A newly constructed bFGF release system with adequate release kinetics may solve these problems. A bFGF-encapsulating hydrogel not only served as a drug carrier, but also effectively preserved fibroblast phenotypes so that the pro-angiogenesis and anti-fibrotic functions of CFs were maintained 39. Additionally, some cardioprotective molecules associated with CFs have been discovered, for example, a novel agent, NM922, of therapeutic value could inhibit the conversion of the CF phenotype in a murine TAC model by preventing the activation of several profibrotic pathways (including pathways involving mTOR/STAT3/E4-BP1, FAK-Akt-P70S6K, and the generation of COX-2) 40. Administration of sacubitril/valsartan (SAC/VAL) appears to be a promising strategy to improve pressure overload-induced cardiac fibrosis by directly acting on fibroblasts. The positive effect of SAC/VAL is mediated by the restoration of protein kinase G (PKG) signaling in CFs 41. In addition, exchange protein activated by cyclic AMP 1 (EPAC1), a signaling molecule activated by adrenergic stimulation, was downregulated in CFs from atrial fibrosis hearts. Overexpression of EPAC1-signaling seemed to be cardioprotective 42. Delivery of ECM biomaterials is another effective strategy to promote cardiac regeneration and maintain heart function recovery 43. Direct cardiac reprogramming offers a potential novel approach for restoring cardiac function, during which CFs could be converted into induced CM-like cells (iCMs) without first reverting them into stem cells 44. Direct cardiac reprogramming can be achieved by the transduction of various cardiac-specific factors 45. A large number of studies have discovered the effectiveness of the direct reprogramming of murine CFs into iCMs *in vitro* 46-50. Importantly, Nam et al. 51 have successfully determined the optimal combination of cardiac transcription factors required to direct the reprogramming of human CFs, including Hand2, GATA4, T-box5, myocardin, miR-1 and miR-133; afterward, the stimulated CFs can be directly reprogrammed to become CMs after the induction of the expression of these proteins and microRNAs, and these human CFs exhibited sarcomere-like structures and calcium transients along with expression of cardiac genes. A recent *in vitro/vivo* study revealed that a combination of TGF-β and WNT inhibitors markedly enhanced the efficiency of GMT (Gata4, Mef2c, and Tbx5)-induced direct cardiac reprogramming, providing robust evidence of the possibility for cardiac regeneration 52.

In summary, therapies for cardiac remodeling by the direct/indirect targeting of CFs via known signaling pathways and molecular mechanisms of CFs are emerging rapidly. The efficacy and safety of these potential therapies require further clinical investigations.

## Immune cells

Immune cells are important parts of the innate immune system of mammals. Research has indicated that acute inflammatory signals stimulate the cardiac regenerative response in neonatal mice 53. Numerous experimental studies have found that the immune response in injured heart tissue helps regulate CM function and induce adverse cardiac remodeling 54-56. The important roles of inflammatory cells, innate immune molecules and the reprogramming of relevant signaling pathways in cardiac remodeling following ischemic heart diseases were reviewed previously 57-59. In this section, we will summarize the very recent advances in the understanding of the effects of heterogeneous populations of immune cells (including macrophages, neutrophils, lymphocytes and mast cells) and regulatory mechanisms on the development of cardiac remodeling.

### Cardiac Macrophages

Macrophages are found in almost all types of tissues. Cardiac macrophage, as an important part of the mononuclear phagocyte system, constitutes approximately 7% of the nonmyocytes in the adult mouse heart 60. Cardiac macrophages are mainly derived from blood-derived monocytes and the yolk sac (YS) 61. Of the two sources, a growing body of studies has paid more attention to monocyte-macrophage transition because of its universality, heterogeneity and complexity. Immature cells in the bone marrow are regarded as significant contributors to blood-circulating monocytes, which constantly migrate to the peripheral tissues, where they differentiate into macrophages 62. Monocytes and macrophages are intimately related in terms of their sources as well as their functions and serve as critical contributors to the innate immune response to inflammation.

#### Heterogeneity of cardiac monocytes and macrophages

Monocytes usually originate from myeloid progenitors in the bone marrow. In mice, monocytes are classified into two subsets, the classical Ly-6C^high^CCR2^high^CX3CR1^low^ and nonclassical Ly-6C^low^CCR2^low^CX3CR1^high^ subtypes, based on Ly-6C expression levels; the former subtype migrates to the infarcted areas and serves as the main contributor to inflammation, while the latter always persists in the circulation and is responsible for maladaptive remodeling 63-65.

In cardiac macrophages, the expression of CCR2 (chemokine receptor type 2, a receptor of CCL2/MCP1 and CCL7/MCP3) greatly influences cellular behaviors. Both inflammatory monocyte-derived CCR2^+^ and tissue-resident CCR2^-^ macrophages are present in mouse hearts. Tissue-resident macrophages (TRMs) are a group of cells that take residence in various tissues (including the heart, gut, and dermis) prior to birth and maintain themselves locally throughout adulthood by performing immune hemostatic and sentinel functions 66, 67. TRMs originate from a transient hematopoietic wave of erythromyeloid progenitors that emerge from the YS, and importantly, TRMs can also be continuously repopulated through self-renewal, completely or partially independent of bone marrow contributions 67, 68. CCR2-positive macrophages could recruit monocytes in an MYD88 (myeloid differentiation primary response 88)-dependent manner, and MYD88, as a key mediator, could further lead to the release of MCPs (monocyte chemoattractant proteins) and the migration of monocytes to the infarcted zone. Selective removal of CCR2^+^ or CCR2^-^ macrophages before MI had conflicting results with regard to monocyte recruitment, LV function and remodeling 69. Similarly, a recent investigation of macrophage heterogeneity in the human heart suggested that the human heart contained distinct subsets of CCR2^+^ and CCR2 ^-^ macrophages. The two subtypes had different origins, localizations, and functions. CCR2^-^ macrophages, which developed during the embryonic stages, tended to appear in viable myocardium and had the potential to orchestrate tissue repair, whereas CCR2^+^ macrophages, which originated from monocytes, preferred to remain in fibrotic sites and were related to inflammation 70. Another discovery regarding cardiac macrophage heterogeneity was reported by Sarah A Dick and colleagues: by utilizing genetic fate mapping and single-cell RNA sequencing (scRNA-seq), the group discovered that healthy hearts contained four kinds of macrophages, TIMD4^+^LYVE1^+^MHC-II^lo^CCR2^-^, TIMD4^-^LYVE1^-^MHC-II^hi^CCR2^-^, and two CCR2^+^MHC-II^hi^ subsets of macrophages, which were independently, partially, and fully renewed by monocytes, respectively. Diversification of these subgroups occurred immediately after MI. The TIMD4^+^ and TIMD4^-^ subgroups were reduced in the early phase post MI but gradually increased via self-renewal 71. ^68^Ga-DOTA-ECL1i, a PET radiotracer, could specifically bind to CCR2^+^ monocytes/macrophages, and the signal was correlated with CCR2^+^ abundance, which provided an effective molecular imaging method to noninvasively visualize the recruitment of monocytes and macrophages to the infarct heart 72.

In conclusion, these findings showed that macrophages are extremely heterogeneous cells present in both human and murine cardiac tissues. Cardiac macrophages can be broadly divided into two subpopulations: cardiac resident macrophages and monocyte-derived macrophages. Resident macrophages, derived from the YS and self-renewal, reside in the myocardium and contribute to the maintenance of the cardiac steady state. In contrast, infiltrating macrophages are continuously repopulated by blood-circulating monocytes in a CCR2-dependent manner after various pathological stimuli, and CCR2^+^ monocyte-derived macrophages are responsible for the inflammatory response to cardiac injuries. However, current knowledge of macrophage subsets is very limited, and a more well-resolved and nuanced understanding of these cells will enable the targeting of disease-promoting macrophage functions.

#### Activation and polarization of macrophages in cardiac remodeling

Macrophages, which are equipped with various signaling receptors, are a crucial cell type that integrates signaling pathways and actively responds to disease-related stress. In healthy hearts, a group of macrophages of both YS and monocyte origin were present 66, 73. In cardiac tissue undergoing infarction, the cellular response differed in different regions. At the site of MI, due to the destruction of resident cells, large numbers of circulating monocytes quickly entered the myocardium and transformed into macrophages, while in the peri-infarct zones, the continuous proliferation of TRMs and monocyte infiltration were both beneficial to the rapid expansion of macrophages; in the remote regions, macrophage populations were supplemented by monocyte recruitment rather than by TRMs 61, 63. Importantly, macrophages residing in the pericardial space near the site of injury displayed cardioprotective functions. A study reported that a large number of Gata6^+^ pericardial cavity macrophages (GPCMs) invaded the pericardial cavity in experimental MI mice. During the progression of remodeling, GPCMs lost the ability to express Gata6 and surprisingly exhibited antifibrotic effects. In line with these findings, depletion of the GPCMs or removal of the pericardial cavity led to a reduction in post MI cardiac remodeling. In addition, GPCMs were found in the pericardial fluid in humans, suggesting that they could be targeted by novel therapies for various heart diseases 74.

**Figure 2:**
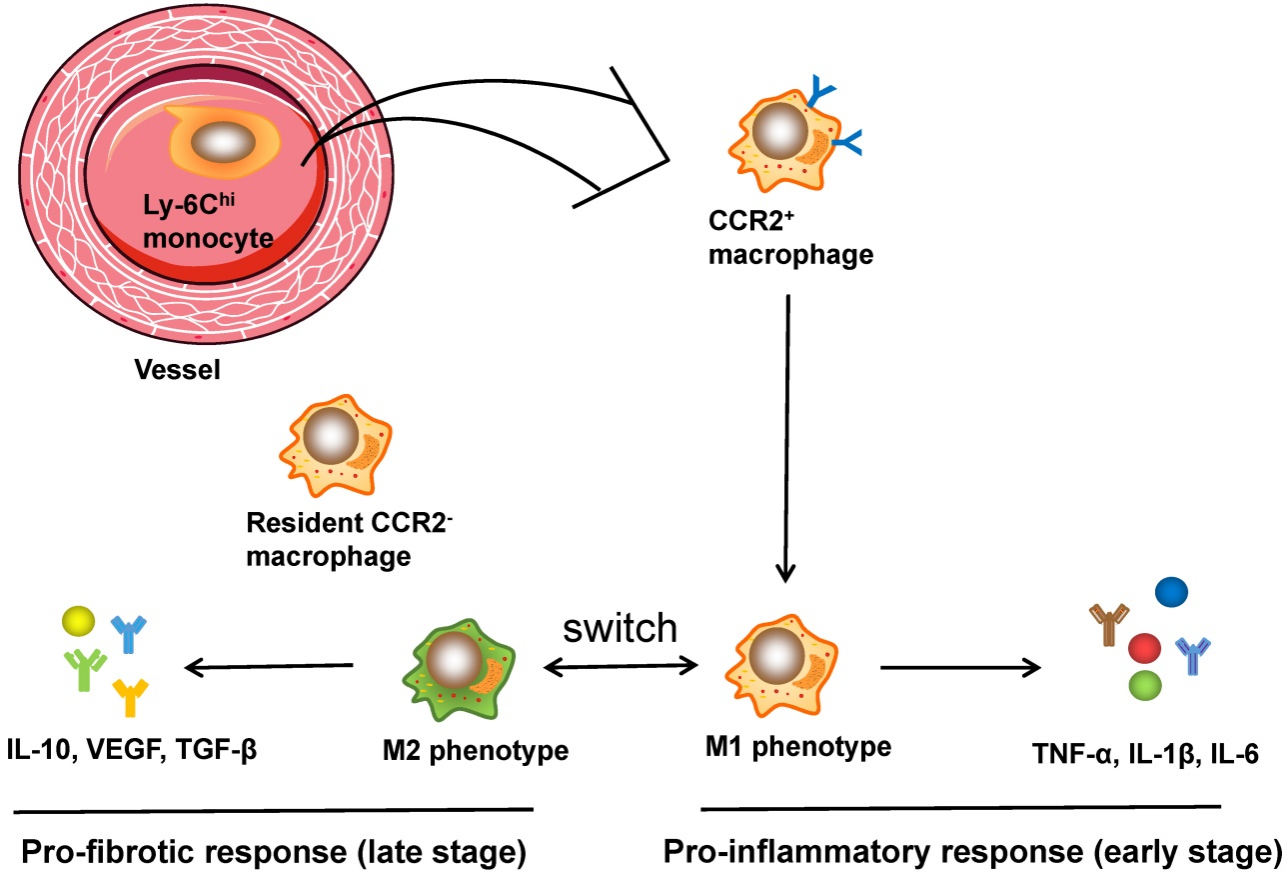
The activation of macrophages in cardiac injuries. CCR2^+^ macrophages, derived from circulating monocytes, have the potential to orchestrate the inflammatory phase and polarize into M1 and M2 phenotypes. Ly6C^hi^ M1 macrophages are characterized by secretion of TNF-α, IL-1β, and IL-6, which are pro-inflammatory cytokines that assist in acute inflammatory response, while Ly6C^lo^ M2 macrophages express and release anti-inflammatory cytokines like VEGF and TGF-β, contributing to CF activation, angiogenesis and wound healing. Moreover, CCR2^-^ macrophages, originating from embryo, reside in the myocardium and are related to fibrogenesis.

It is well established that the function of cardiac macrophages is largely dependent on the two subpopulations, comprising pro-inflammatory M1 and reparative M2 macrophages 75. Ly6C^hi^ M1 macrophages were characterized by the secretion of TNF-α, IL-1β, and IL-6, which are pro-inflammatory cytokines that contribute to the acute inflammatory response. In contrast, Ly6C^lo^ M2 macrophages express and release anti-inflammatory cytokines such as VEGF and TGF-β, contributing to CF activation, angiogenesis and wound healing 76. In addition, infusion of IL-10* in vivo* significantly increased the expression of M2 marker genes along with CF activation (as evidenced by CF proliferation, recruitment and ECM accumulation), and ultimately led to the improvement of cardiac remodeling both structurally and functionally 77. A recent study using flow cytometry and RNA sequencing technology successfully mapped the transcriptomes of polarized macrophage during the first week post MI 78: within the first day post MI, macrophages showed the unique features of pro-inflammatory proprieties and ECM degradation, while the macrophages on day 3 displayed the upregulation of proliferation and phagocytosis as well as the reprogramming of metabolism; macrophages on days 7 exhibited proreparative signatures characterized by the elevation of Col1a1, Col3a1, and postn expression. These data demonstrate the immediate responses and continuous trans-differentiation of the phenotypes of macrophage from an inflammatory M1 phenotype to a reparative M2 phenotype post MI and during tissue remodeling. In conclusion, cardiac macrophages play leading roles in regulating heart remodeling and show dynamic spatial and temporal changes. Figure 2 illustrates the activation process of cardiac macrophages.

#### Potential therapies targeting macrophages

Preclinical research has revealed a possible diagnostic macrophage biomarker, the scavenger receptor stabilin-1. Stabilin-1^+^ macrophages accumulated and peaked in number in the regenerative phase in both infarct and peri-infarct sites and remained unchanged in the late stage 79. Moreover, many recent studies have suggested potential treatments for cardiac remodeling that target macrophage polarization. Long-term administration of eicosapentaenoic acid (EPA), which is known to reduce the incidence of nonfatal coronary diseases, especially reduced remodeling by inhibiting macrophage polarization towards an M1 phenotype 80. Aminooxyacetic acid (AOAA) remains another novel agent. Short-term administration of AOAA led to a decreased proportion of M1 macrophages, an elevated proportion of M2 macrophages, and inactivation of the NLRP3-caspase1/IL-1 beta pathway 81. In addition, some other agents such as hemin-carried drugs based on lipid 82, NADPH oxidase 4 (Nox4) 83, hyaluronic acid oligosaccharides (o-HA) 84, played possible beneficial roles in restoring cardiac function post MI by targeting cardiac macrophages and skewing them towards an M2 anti-inflammatory phenotype, which provided a novel strategy to regulate inflammation, reduce adverse remodeling, and improve the contractile function of infarcted hearts. Interestingly, some commonly used hypoglycemic drugs had certain effects in ameliorating cardiac remodeling. By modulating M2 polarization and preventing myofibroblast infiltration, the selective SGLT2 inhibitor dapagliflozin attenuated post MI remodeling in rats 85. Similarly, in preclinical animal experiments, evidence has shown that nanoparticle (NP)-based agent pioglitazone is a potential antagonist of cardiac remodeling. The NPs were delivered specifically to cardiac monocytes/macrophages, reducing the recruitment of macrophages and promoting the polarization of macrophages to the M2 phenotype 86. Interestingly, a recent study revealed that stem cell therapy could dramatically improve cardiac function in mice after ischemia-reperfusion injury; mechanistically, cell therapy triggered an acute immune response characterized by temporal and regional accumulation of CX3CR1^+^ and CCR2^+^ macrophages 87. Transplantation of murine neonatal macrophages has also been shown to be effective for adult cardiac repair 88. Overall, given the biphasic response of cardiac macrophages to heart injuries, quite a few therapies targeting cardiac macrophages have been shown to be cardioprotective mainly by directly modifying macrophages or promoting macrophage phenotypic switching from a pro-inflammatory to an anti-inflammatory subtype.

### Neutrophils

Neutrophils are the prominent leucocytes, accounting for approximately 50-70% of circulating leukocytes in humans and 10-25% in mice 89. As an important component of the innate immune system, neutrophils act as a key player in cardiac remodeling induced by various external or internal stimuli.

#### Activation of neutrophils in cardiac remodeling

Neutrophils, which initiate the inflammatory reaction, were traditionally considered as “bad cells” post MI. Stimulated by cell debris, danger-associated molecular patterns (DAMPs) and cytokines from neighboring cells, a large number of neutrophils quickly migrate and accumulate in the injured areas a few hours after infarction, where they generate reactive oxygen species (ROS) and release granule contents, eventually causing acute tissue injuries 89, 90. The elevation of neutrophils usually implies poor outcomes in patients suffering from acute coronary syndrome 91.

Due to the improved understanding of neutrophils, it has been recently appreciated that neutrophils modulate cardiac remodeling through positive effects. Cardiac neutrophils also undergo polarization; as previously reported, they often consisted of the N1 (expressing CCL3, IL1β, and TNFα) and N2 phenotypes (expressing IL10 and CD206) 92. By performing aptamer proteomics, researchers have successfully mapped the polarization of cardiac neutrophils in coronary artery ligation-induced MI mouse models. Neutrophils on day 1 after MI were characterized by a high level of degranulation, the initiation of inflammation and the breakdown of ECM by activating matrix metalloproteinase (MMP); on days 3, the cells exhibited the upregulation of apoptotic signaling, cathepsin activity, and ECM reorganization; on day 5, neutrophils showed further reconstruction of the ECM and resolution of inflammation; and neutrophils on day 7 had typical reparative profiles, with high expression of galectin-3, fibronectin, and fibrinogen 93. Therefore, it is apparent that neutrophils are extremely complex and capable of a variety of regulatory functions. The contributions of this cell type result from both its “pro-inflammatory” and “healing” effects.

#### Important roles of NGAL

Neutrophil gelatinase-associated lipocalin (NGAL), a kind of lipocalin first discovered in neutrophils and approved derived from various types of immune cells, remarkably increased in abundance within several days after MI as well as in clinical and experimental HF 94, 95. NGAL blockade *in vivo* showed beneficial effects on limiting cardiac inflammation and fibrosis. The study also demonstrated that NGAL was mineralocorticoid receptor (MR)-dependent and exerted its pro-fibrotic roles in experimental MI and cardiac remodeling through the NF-κB signaling pathway 96. In agreement with these results, NGAL was also found to be elevated in aldosterone-induced cardiac injuries, and the inhibition of NGAL led to anti-inflammatory and anti-fibrotic effects 97. Moreover, clinical research has revealed the possibility of the use of NGAL as a supplementary marker for cardiovascular conditions 98. Surprisingly, a recently published study announced the potential cardioprotective roles of neutrophils in cardiac healing, as evidenced by the excessive fibrosis, increased levels of markers for HF, and worsening cardiac function in neutrophil-depleted mice; mechanistically, abundant neutrophils are required to recruit monocytes and promote macrophage polarization into the M2 reparative phenotype, which mainly occurs through the induction of NGAL 99. In summary, these data indicate the pivotal effects of NGAL on the prognosis of patients with HF and the protective role of anti-NGAL strategies.

#### Neutrophil signaling in cardiac remodeling

Another interesting study indicated that neutrophil signaling post MI differed according to gender. Previous clinical investigations have found differences between men and women in terms of clinical manifestations and long-term prognosis after MI 100. Through a combined analysis of retrospective, prospective and translational data, DeLeon-Pennell and coworkers convincingly revealed that sex differences existed in post MI inflammatory effects and wound healing. In neutrophils in males, PPARγ (peroxisome proliferator-activated receptor γ) and LXR/RXR (liver X receptor/retinoid X receptor) served as the main contributors to immunosuppression; while in those in females, thrombospondin and LXR/RXR signaling played critical roles. In aging females, due to the inability to activate LXR/RXR signaling, a large amount of IL-6 and downstream signaling were activated, finally leading to the loss of protection 101. These results provide evidence of sex-related differences in the treatment of male and female patients with cardiac remodeling.

### Lymphocytes

Lymphocytes, which are an important subset of inflammatory cells, are roughly divided into T-lymphocytes and B-lymphocytes according to the organs where they differentiate, develop and mature. Both cell types participate in the modulation of cardiac remodeling 102-104. Traditionally, CD4^+^ and CD8^+^ cells are two major categories of T cells that are identified by their cytokine secretion as well as their immune effects, and indeed, the former plays a predominant role in cardiac remodeling despite the etiology 105. CD4^+^ cells consist of heterogeneous subsets, including INF-γ-producing T-helper (Th)1 cells, IL-4-expressing Th2 cells, IL-17-positive Th17 cells and regulatory T cells (Tregs), among which the Th1 and Th17 subpopulations are pro-inflammatory, while Th2 and Tregs cells are anti-inflammatory 102.

#### Engagement of T-lymphocytes in cardiac remodeling

In heart tissue injured by pathological stimuli, cell debris and released antigens are delivered by dendritic cells (DCs) to T lymphocytes; subsequently, T cells become responsive to these pathogens and are broadly activated. Recruitment of CD4^+^ cells from the circulation to sites of injury requires sustained induction by cytokines, chemokines and adhesion molecules 106. C-X-C motif chemokine ligand 12 (CXCL12) and its receptor, CXCR4, are regarded as the main effectors mediating inflammatory cell recruitment and activation in injured tissues 107; other molecular mediators include CXCL9, CXCL10, CXCL11, CXCR3, ICAM-1, and CCR5 108. Recently, published studies have revealed the remarkable expansion of both CD4^+^ and CD8^+^ T-lymphocytes; the Th1, Th2, Th17, and Treg CD4^+^ subgroups were present in the circulation, HF myocardium, spleen and lymph nodes in murine models of ischemic HF, with the Th2 and Th17 subsets being predominant 109. Consistent with these findings, T-cell infiltration was observed in peripheral blood and heart samples obtained from ischemic HF patients. By performing deep sequencing, researchers uncovered clonal expansion of the T-cell receptor repertoire in IHF, and these T cells were characterized by enhanced proliferation and memory- and effector-like properties 110.

Among various T cell types, Tregs expressing transcription factor forkhead box protein 3 (Foxp3) are a well-characterized CD4^+^ subset that is typically immune-suppressive and helps maintain the stability of the immune system 111. Correspondingly, Tregs had a positive influence on post MI healing, mainly during the transition from the inflammatory phase to the resolution phase 112. Large numbers of CD4^+^CD25^+^Foxp3^+^ Tregs were diffusely expanded and activated in the heart and other organs after ischemic cardiomyopathy 113, 114. These “pathological” Tregs, which are different from those found in steady states, displayed pro-inflammatory properties (with the expression of TNFR1, TNFα and INF-γ) along with anti-angiogenetic and pro-fibrotic features. Selective ablation of these dysfunctional Tregs, interestingly, greatly alleviated cardiac fibrosis and hypertrophy, reversed remodeling and improved LV systolic function 115. Hence, restoration of the normal features of Tregs remains the basis of maintaining “homeostatic” function. In conclusion, these findings indicate the significant effects of T cells on heart remodeling. Nevertheless, the details of T cell-specific antigens still need to be investigated. In addition, the respective roles of different subpopulations of T cells need to be identified.

#### Engagement of B-lymphocytes in cardiac remodeling

The roles of B lymphocytes in cardiac remodeling triggered by heart injury remained unrecognized for a long time. In Ang II-induced HF models, B-cell depletion exhibited cardioprotective effects, as evidenced by the significant reduction of cardiac hypertrophy and fibrosis, while B-cell reconstitution had the opposite effects 116. After diphtheria toxin (DT)-induced acute heart injury or I/R injury, cytometry analysis revealed an increase in CD19^+^CD11b^-^ B lymphocytes in comparison to that in naïve hearts, and further *in vitro* experiments demonstrated that the activation of B cells occurred in a TIRAP (Toll-interleukin 1 receptor-domain containing adaptor protein)-dependent manner 117. However, since much is still unknown about B cells (including antibodies and subsets of B cells), effective agents to alleviate heart remodeling specifically by modulating B lymphocytes remain to be investigated.

#### Potential treatments targeting lymphocytes

Therapies that affect lymphocyte dynamics (recruitment, proliferation and activation) and relevant signaling are seemingly promising strategies. Experimental data revealed that a highly selective inhibitor of CXCR4 enhanced tissue healing and cardiac function after MI via cardiac mobilization of spleen-reserved CD4^+^Foxp3^+^ Tregs due to their specific immunoregulatory function 107. In addition, due to an inadequate understanding of Treg clinical applications, therapies often focus on the indirect effects of this cell type. Tolerogenic dendritic cells (tDCs), a potential mediator of infarction healing, play therapeutic roles by modulating Tregs and M1/M2 macrophage polarization 118. Local intravenous injection of tDCs could improve the immune microenvironment, resulting in enhanced wound healing and improved survival after MI. Another newly declined immunotherapy using engineered T cells has provided novel insights into the treatment of cardiac fibrosis; cardiac injury and ovalbumin peptide (OVA) expression in CFs were induced by Ang II infusion and Cre recombinase administration, respectively. One week later, CD8^+^ OT-I T cells (CD8^+^ T cells expressing a cognate T cell receptor against OVA) were transformed. The *Postn^MCM^;RosaOVA* mice that received modified T cells exhibited lower levels of fibrosis and remodeling 119. Hence, applicable therapies are still limited, and methods of transforming “pathological” lymphocytes into normal lymphocytes and repressing the lymphocyte-specific inflammation are awaiting development.

### Mast Cells

Mast cells (MCs), which are morphologically equipped with numerous cytoplasmic granules, are one of the most well-known cell types that are resident in almost all organs in response to allergy. In fact, MCs are multifunctional cells that participate in a large range of pathophysiologic processes in addition to allergic responses 120. The function of MCs is dependent on various regulatory factors, including pro-allergic (histamine, TNF, and proteases), pro-inflammatory (INF-γ, IL-1β and IL-6), immunosuppressive (IL-10 and IL-13), pro-fibrotic (TGF-β1 and bFGF) and anti-fibrotic (IL-33, VEGF and PGD2) mediators 120, 121. Immediately after heart injury, MCs in the heart quickly responded to DAMPs and were significant mediators of cardiac remodeling. The regulation and dual roles of MCs in adverse myocardial remodeling have been reviewed by Scott P. et al. 122.

#### Recent findings regarding MCs in cardiac remodeling

Anta Ngkelo and colleagues have investigated the roles of MCs in LV remodeling. Flow cytometry/imaging analysis revealed that MCs significantly expanded on day 7 after MI surgery, and MC-specific gene expression occurred in coordination with the release of typical granule contents. In the damaged heart, the infiltration of MC progenitors was observed. To further evaluate the function of MCs in infarcted myocardium, MC-deficiency mice were generated by Cre-mediated MC eradication (Cpa3^cre/+^), and it was found that the contractile function of CMs (based on the LV wall thickness, LV postsystolic diameter, interventricular septal end-diastolic diameter, and shortening fraction) was decreased in MC-deficient mice compared to that in WT mice. In terms of the involved mechanism, the cardioprotective effects of MCs resulted from MC-mediated enhanced myofilament sensitization to Ca^2+^ 123. MCs are also involved in the progression of right ventricle (RV) remodeling. An increased number of MCs infiltrated and became activated in the RV tissue in mice generating pulmonary artery banding (PAB). The proportion of degranulated MCs started to increase at day 3 after PAB and peaked at D7; in contrast, the MC density began to increase at 2 weeks and reached a plateau by the third week after surgery. Notably, the levels of the MC marker c-Kit and MC-specific mMCP in RV were significantly elevated 124. Thus, MCs may represent an evaluable target for new therapeutic approaches for both LV and RV remodeling.

Notably, among the granule contents released by MCs, chymase is one of the most important components. Among all mouse chymases (consisting of mouse mast cell protease-1, -4, -5, and -9), mouse mast cell protease-4 (mMCP4) is most similar to that in humans 125. An increasing number of studies have reported that mMCP4 participates in many CVDs and even HF. mMCP4 was extensively expressed in MCs post MI, and the deficiency of mMCP4 played a protective role in murine cardiac function after coronary artery ligation, which was characterized by reduced CM apoptosis, reduced accumulation of immune cells, impaired TGF-β signaling, enhanced ECM degradation, and amelioration of the ejection fraction 126. Similarly, another finding demonstrated that in mMCP-4 knockout mice, the size of the infarcted area, ventricular remodeling, and apoptotic signaling were prominently reduced compared to those in the WT congeners 127. Insulin-like growth factor-1 (IGF-1), a protective polypeptide, was degraded by mMCP4. Genetic deletion of Mcpt4 (the gene encoding mMCP4) reduced the infarction sizes and improved cardiac function via decreased degradation of IGF-1 128. These data firmly indicate the possible therapeutic roles of chymase blockage in post MI remodeling.

Sex differences in MC regulation between males and females have also been discovered. It has been verified that estrogen is capable of inhibiting pressure overload-induced cardiac remodeling. Similarly, after TAC surgery, ovariectomized (OVX) rats displayed more serious LV interstitial fibrosis and cardiac hypertrophy than sham-operated rats. Estrogen supplementation in TAC-OVX rats attenuated cardiac adverse remodeling by preventing MC chymase synthesis and release 129. In accordance with this finding, administration of the mast cell stabilizer cromolyn sodium could mitigate changes in the wall thickness and LV mass and cardiac fibrosis in OVX female mice, providing evidence that MC antagonists are promising for the treatment of estrogen loss-induced LV remodeling (especially menopausal women) 130. In summary, MCs are key cells modulating cardiac remodeling under a sex-dependent mechanism. The clinical translation of research of sex differences in MCs may provide new insights into the treatment of patients of different genders.

## Endothelial cells

Cardiac endothelial cells (ECs) are simple squamous epithelial cells present in the inner surface of the heart and blood vessels. ECs accounted for 64% of non-CMs in adult mice, outnumbering other cell types 60. Genetic lineage tracing of cardiac resident cells by detecting stem cell antigen-1 (Sca-1) has uncovered that in mouse models of MI, Sca-1^+^ cells are a significant source of vascular endothelial cells, which accumulate heavily in heart tissue and quickly respond to ischemic stimuli 131. In recent research, RNA sequencing technology was utilized to assess the transcriptome features of endothelial cells in cardiac remodeling. The data showed that 55 fetal genes were expressed in LAD-ligated hearts; the downregulated genes included *Nbl1, Myoz2, Lbh, Gfra1, Gpx3* and *Efemp*, while the upregulated genes were *Tnc, Anp32b, Thy1, Lpar4 and Car3* 132. ECs participate in controlling CM contraction function as well as wound healing after pathological stress by secreting various biologically active substances, such as adhesion molecules (ICAM-1 and tenascin-C), pro-fibrotic cytokines (endothelin-1, Ang II, TGFβ1, periostin, and connective tissue growth factor), pro-inflammatory mediators (IL-6 and IL-1β), pro-angiogenetic factors (VEGF, PLGF, PDGF, bFGF, EGF, and HGF), cardioprotective mediators (follistatin-like 1, IGF-1, dickkopf-3, apelin, and LIF), some CXC chemokines, and other effectors 133-135.

## Pericytes

Pericytes, which are also known as Rouget cells or parietal cells, are cells that surround ECs in capillaries and microvessels. Pericytes are identified by the expression of CD146, PDGFR-β, and alkaline phosphatase. The modulatory roles of pericytes are poorly understood. Previous studies have confirmed the potential reparative effects of pericytes. Combined delivery of pericytes and cardiac stem cells into the infarcted heart surprisingly improved cardiac contractility, as shown by echocardiographic, histological and molecular evaluations 136. Moreover, in a swine model of AMI, transplantation of allogenic swine adventitial pericytes resulted in pro-angiogenic as well as anti-fibrotic outcomes 137.

## Conclusions

**Figure 3:**
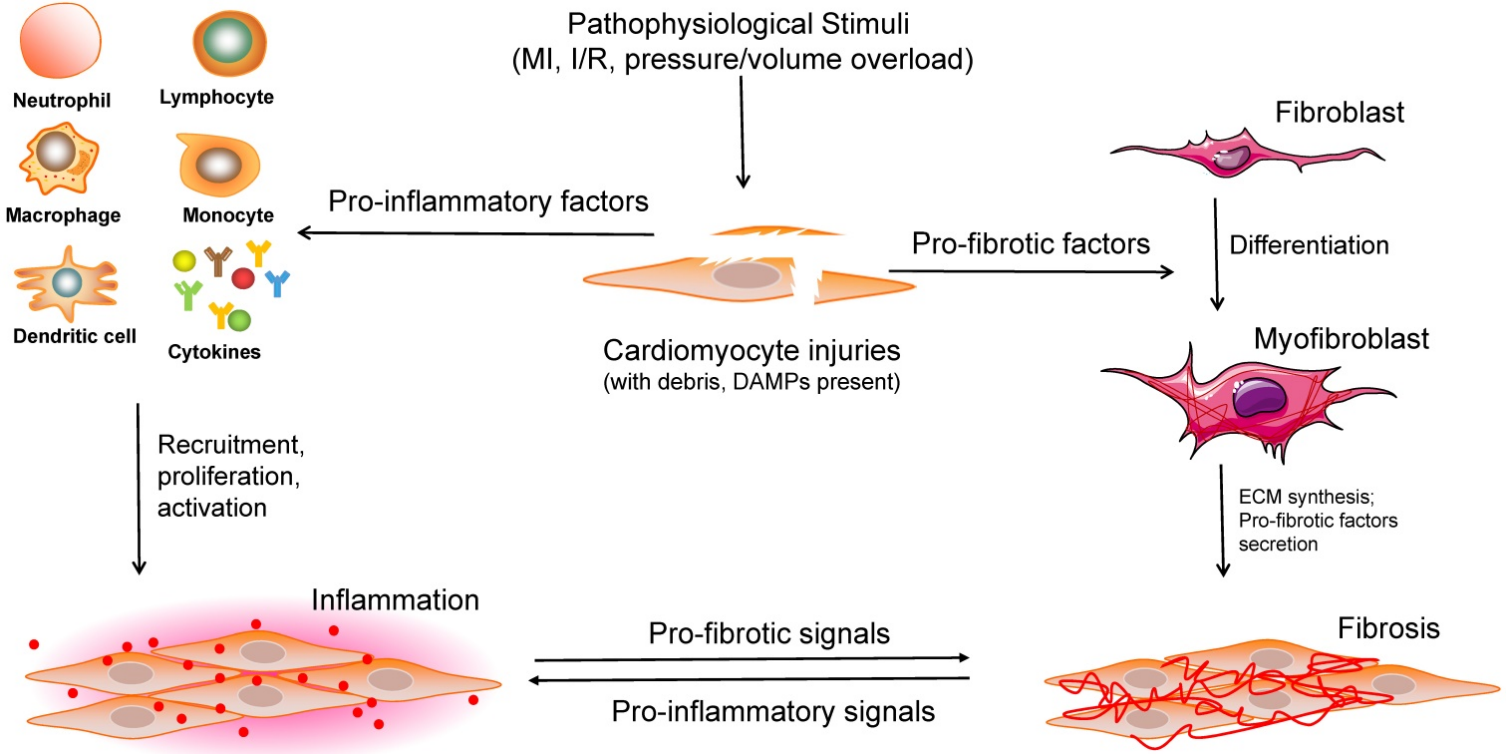
Overview of the interactions between cardiomyocytes and noncardiomyocytes in the process of cardiac remodeling. When stimulated by various pathophysiological insults, large numbers of cell debris, regulatory factors, and DAMPs are expanded in the injured heart tissue. Subsequently, a range of immune cells are recruited, proliferated and activated to induce inflammation. Moreover, pro-fibrotic factors contribute to fibroblast-to-myofibroblast transition, ECM synthesis, pro-fibrotic cytokines secretion, and cardiac fibrosis. DAMPs: damage-associated molecular pattern molecules, ECM: extracellular matrix.

Cardiac remodeling, which was once regarded as a simple outcome induced by an increased workload, has recently been shown by a growing number of studies to be a complex response to various cardiac pathophysiological stimuli, involving a range of infiltrating and activated cells (including CMs, a variety of inflammatory cells, and other non-CMs). Considering that the process of cardiac remodeling is roughly divided into the inflammatory and fibrotic phases, the significant roles of CFs and immune cells must be emphasized (Figure 3). It is of great value to understand the different cell phenotypes and their activation mechanisms in response to internal or external pathological stimuli in the heart. Although several therapies targeting different non-CMs have been reported recently (Table 1), a more adequate and comprehensive understanding of regulatory cell types is required for the development of potential clinical interventions.

With the wide use of selective depletion and conditional gene knockout methods, the effects of certain cells in cardiac remodeling have been gradually revealed. However, until now, most research has focused on MI murine models and ignored the cellular phenotypes and potential signaling pathways involved in other CVDs. In addition, single studies often focus on the effects of one specific cell type in a certain phase, and studies that simultaneously explore multiple cell types and their interactions that contribute to cardiac remodeling are very rare. Importantly, although many animal studies have revealed detrimental or protective effects of non-CMs on tissue remodeling, preclinical or clinical investigations are still lacking, and wide gaps exist between the bench and the bedside. In view of the heterogeneity and complexity of non-CMs in the regulation of cardiac remodeling, deeper exploration is urgently needed to dissect the cell biology and to find new therapeutic strategies; furthermore, an improved understanding is required to identify the patients who may benefit from the new findings.

## Figures and Tables

**Table 1:** Recent advances in potential therapeutic approaches targeting non-CMs in cardiac remodeling

Targeted cells	Potential therapy	Species	Disease model	Key observations	Mechanism
Cardiac fibroblasts	NM922 40	mouse	pressure overload-induced cardiac hypertrophy and HF	attenuated LV dilation and hypertrophy, inhibited fibroblast activation	reducing the activation of FAK-Akt-P70S6K and STAT3/E4-BP1 pathway
	LCZ696 41	mouse	left ventricle pressure overload-induced cardiac remodeling	ameliorated pressure overload- induced cardiac fibrosis	restoration of PKG (protein kinase G) signaling
	direct cardiac reprogramming 51	human	-	human adult fibroblasts were reprogrammed into iCMs, along with expression of cardiac markers, and sarcomere-like structures	inducement of cardiac transcription factors and muscle-specific miRNAs
	oleic acid 138	mouse	Ang II-induced cardiac remodeling	prevented Ang II-induced cardiac fibrosis and improved heart function	suppressing the expression of FGF23 (fibroblast growth factor 23)
	DM-celecoxib 139	rat	isoprenaline-induced cardiac remodeling	suppressed cardiac hypertrophy and fibrosis	inhibiting Akt-mediated GSK-3 phosphorylation
	simvastatin 140	human	TGF-β1-induced human ventricular fibroblast differentiation	reduced hVF proliferation and myofibroblast differentiation	activation of protein-phosphatases PPM1A and PP2A interacting with SMAD2/3
	Givinostat 141	mouse	acute myocardial infarction generated by LAD ligation	reduced cardiac fibrosis and improved cardiac performance	targeting endothelial-to-mesenchymal transition (EndMT)
Cardiac macrophages	aminooxyacetic acid 81	mouse	cardiac remodeling after LAD ligation-induced MI	attenuated post-MI cardiac remodeling	balancing M1/M2 macrophage phenotypes and inhibiting NLRP3-Caspase1/IL-1β pathway
	eicosapentaenoic acid 80	mouse	cardiac remodeling after LAD ligation-induced MI	attenuated post-MI cardiac remodeling	inhibiting macrophage polarization toward pro-inflammatory M1 phenotype
	hemin/HA-LP 82	mouse	post-MI cardiac remodeling	improved infarct-related regional function and promoted infarct healing	switching macrophages toward M2 anti-inflammatory phenotype
	dapagliflozin 85	rat	post-MI cardiac remodeling	attenuated cardiac fibrosis	regulating macrophage phenotype through RONS/STAT3-dependent pathway
	pioglitazone-NPs 86	mouse	post-MI cardiac remodeling	attenuated cardiac remodeling	reducing macrophage recruitment and polarizing macrophages towards the pro-healing M2 phenotype
	cardiac stem cell therapy 87	mouse	ischemia-reperfusion injury	enhanced cardiac function	through an acute immune response, characterized by a significant accumulation of CCR2^+^ and CX3CR1^+^ macrophages
	transplantation of neonatal cardiac macrophages 88	mouse	post-MI cardiac remodeling	improved MI-injured adult cardiac repair	stimulating the proliferation of CMs
	Qishen Granule 142	rat	cardiac remodeling after LAD ligation-induced MI	attenuated myocardial fibrosis	suppressing the recruitment of monocytes via MCP1/CCR2 pathway, and balancing M1/M2 macrophage phenotypes
	miRNA-21 NPs 143	mouse	cardiac remodeling after LAD ligation-induced MI	reduced hypertrophy, fibrosis and cell apoptosis	specifically targeting macrophages and eliciting their phenotype switch from M1 to reparative M2
Neutrophils	memantine 144	rat	isoproterenol-induced HF	reduced cardiac remodeling and improved cardiac performance	reducing lipid peroxidation and neutrophil infiltration
Lymphocytes	tolerogenic dendritic cells 118	mouse	cardiac remodeling after LAD ligation-induced MI	improved cardiac remodeling, preserved left ventricular systolic function, and improved survival	inducing a systemic activation of MI-specific Treg cells
	CD8^+^ OT-I T cells 119	mouse	Ang II/PE-induced cardiac fibrosis	attenuated myocardial fibrosis and hypertrophy	directly modifying T cells
	rituximab 145	mouse	pressure overload-induced cardia remodeling	suppressed myocyte hypertrophy, fibrosis and oxidative stress, and improved heart function	inhibiting pro-inflammatory cytokines and Th2 cytokine-mediated IgG production from B cells
Endothelial cells	serelaxin 146	mouse	cardiac fibrosis induced by ascending aortic constriction (AAC) and Ang II infusion	attenuated myocardial fibrosis in both models	preventing EndMT through the endothelial Relaxin family peptide receptor 1
	VEGF nanoparticles 147	mouse	post-MI cardiac remodeling	increased vascular density in the peri-infarct region, and improved the LV contractile function 4 weeks after treatment	promoting neovascularization in the infarcted heart
	VEGF-B gene therapy 148	mouse	doxorubicin-induced cardiomyopathy	reduced whole-body wasting, and improved pathological remodeling,	protecting endothelial cells from apoptosis and restoring their normal function
	VEGFA/S1P-deliveried bone marrow cells 149	mouse	post-MI cardiac remodeling	increased endothelial cells, prevented cardiac fibrogenesis and adverse cardiac remodeling	improving micro-vascularization and oxygen diffusion

## Abbreviations

Akt: protein kinase B
bFGF: basic fibroblast growth factor
BMC: bone marrow-derived cell
CCL: chemokine C-C motif ligand
CCR: C-C chemokine receptor
CF: cardiac fibroblast
CM: cardiomyocyte
COX: cyclooxygenase
CVD: cardiovascular disease
CXCL: C-X-C motif chemokine ligand
CX3CR1: CX3C chemokine receptor 1
DAMP: danger-associated molecular pattern
DDR2: discoidin domain receptor 2
EC: endothelial cell
ECM: extracellular matrix
eGFP: enhanced green fluorescent protein
EMT: epithelial-to-mesenchymal transition
EndMT: endothelial-mesenchymal transition
EPAC1: exchange protein activated by cyclic AMP 1
EPDC: epicardium-derived progenitor cell
ERK: extracellular regulated protein kinase
FAP: fibroblast activation protein
Foxp3: forkhead box protein 3
HF: heart failure
ICAM: intercellular cell adhesion molecule
IGF-1: insulin-like growth factor-1
IL: interleukin
LV: left ventricle
Ly-6C: lymphocyte antigen 6C
LYVE1: lymphatic vessel endothelial hyaluronan receptor-1
MAPK: mitogen-activated protein kinase
MC: mast cell
MCP: monocyte chemoattractant protein
MHC-II: major histocompatibility complex class II
MI: myocardial infarction
MK5: MAP kinase-activated protein kinase 5
mMCP4: mouse mast cell protease-4
MMP: matrix metalloproteinase
MRTF: myocardin-related transcription factors
MSC: mesenchymal stem cell
mTOR: mammalian target of rapamycin
MYD88: myeloid differentiation primary response 88
NGAL: neutrophil gelatinase-associated lipocalin
Non-CM: noncardiomyocyte
PAB: pulmonary artery banding
PC1: polycystin-1
PDGF: platelet derived growth factor
PDGFR-β: platelet derived growth factor receptor beta
PKG: protein kinase G
PPARγ: peroxisome proliferator-activated receptor γ
ROS: reactive oxygen species
Sca-1: stem cell antigen-1
SRF: serum response factor
STAT3: signal transducer and activator of transcription 3
TAC: transverse aortic constriction
TGF-β: transforming growth factor-beta
TIMD4: T-cell immunoglobulin and mucin domain containing protein 4
TNFR1: tumor necrosis factor receptor 1
Tregs: regulatory T cells
TRM: tissue resident macrophage
VEGF: vascular endothelial growth factor
YFP: yellow fluorescent protein
YS: yolk sac
